# Contractile properties of superficial skeletal muscle affect postural control in healthy young adults: A test of the rambling and trembling hypothesis

**DOI:** 10.1371/journal.pone.0223850

**Published:** 2019-10-17

**Authors:** Sunghoon Shin, Matija Milosevic, Chul-min Chung, Yungon Lee

**Affiliations:** 1 School of Kinesiology, Yeungnam University, Gyeongsan, Republic of Korea; 2 Graduate School of Engineering Science, Department of Mechanical Science and Bioengineering, Osaka University, Toyonaka, Japan; University of L'Aquila, ITALY

## Abstract

The rambling and trembling analysis separates the center of pressure (COP) fluctuations into two components: rambling (supraspinal contribution) and trembling (muscle stiffness / reflexive properties contribution). We examined whether the trembling component is correlated to the contractile properties (muscle stiffness and contraction time) of lower limb superficial skeletal muscles to experimentally test the rambling and trembling hypothesis. We hypothesized that muscle stiffness and contraction time, would be: (a) more correlated with; and (b) have a greater impact on the trembling component compared to the rambling component. Thirty-two healthy young adults were recruited for the study and tensiomyography was used to assess mechanical muscle responses to a single electrical stimulus to calculate muscle stiffness and contraction time based on radial muscle belly displacement measurements of lower limb muscles unilaterally. Moreover, upright postural control was assessed using a force plate to record ground reaction forces and moments and calculate the COP fluctuations during two 30 seconds trials. From the COP fluctuations, rambling and trembling time series were extracted, and all fluctuation time series were described using a number of different time-domain and frequency-domain parameters in both the anterior-posterior and medial-lateral directions. Our results demonstrated that both muscle stiffness and contraction time were moderately correlated with time-domain and frequency-domain parameters of the trembling component, as compared with those of the rambling component which was not as well correlated. Moreover, they also predicted the trembling component better. Overall, these results imply that postural control during quiet stance is, in part, related to intrinsic muscle stiffness in the lower extremities. Moreover, we showed that the rambling and trembling hypothesis is effective in separating postural sway fluctuations during upright posture to extract the contributions of muscle stiffness / reflexive properties (trembling), and likely the supraspinal contribution (rambling).

## Introduction

Postural control is a complex mechanism in which multiple sensory systems (i.e., visual, vestibular, and somatosensory), muscular activations, and passive dynamics (i.e., ligament and joint stiffness) are coordinated simultaneously within the central nervous system (CNS) [1]. One of the main ways to study postural control is through ground reaction force and torques measured from a force plate during quiet standing. These measurements can be used to calculate changes in the center of pressure (COP), which is the projection point of the body center of mass (COM) resulting from the vertical force acting on the ground and it is considered to reflect the systemic neuromuscular response to COM imbalance [2, 3]. Standing postural control is a combined result of maintaining support against gravity (i.e., COM at a constant height) and postural equilibrium (i.e., COM within the base of support) [4]. However, a limitation of the COP analysis technique is that the changes in postural control are a result of integration of the inputs of various sensory systems, dynamic control of muscles, as well as passive influence of ligaments and musculoskeletal system, which makes it difficult to distinguish the role of each component separately.

Zatsiorsky and Duarte [5, 6] proposed a decomposition method for the COP time series in which the fluctuations are separated into the rambling and the trembling components. The Rambling component is defined as an instantaneous equilibrium reference point (IEP), which is the zero horizontal force point and it represents the slower postural fluctuations of the reference point [4, 5]. The Trembling component is defined as the fluctuation of the COP around its reference point, which represents the faster postural deviations of the body from its reference point [4, 5]. It is proposed that the Rambling component reflects the processes of the CNS that are controlled by the supraspinal centers (i.e., the brain), whereas the Trembling component reflects the peripheral mechanisms of postural control system, such as spinal reflexes and/or passive mechanical properties of the muscles, ligaments and joints [5, 6]. Previous studies demonstrated experimentally that Rambling and Trembling trajectories, separated from the COP fluctuations, reflect these separate components [5–9]. These studies utilized a variety of conditions to manipulate feedback or task constraints, including visual feedback [7], joint fixation [8], and support surface area [9] during upright standing. For instance, Danna-Dos-Santos et al. [7] reported that when an individual needed to track a small visual target of the COP and head movements, the Rambling component decreased and the Trembling component increased, which suggests that dual-tasking decreased voluntary control (i.e., Rambling) component of postural sway. Rambling and Trembling decomposition was also utilized to investigate characteristics of postural control in various neurological / musculoskeletal injury populations. For instance, Bennett et al. [10] investigated Rambling and Trembling fluctuations in adolescents with scoliosis and observed a reduction in both parameters after subjects were asked to reduce their sway, which suggests that scoliosis of adolescents did not change the patterns of sway movements. However, no previous studies directly tested effect of muscle contraction properties on Rambling and Trembling decomposition experimentally. Sosnoff et al. [11] showed a higher relative contribution of Trembling during upright posture in patients with multiple sclerosis with increased spasticity compared to healthy control subjects, which directly demonstrated the contribution of Trembling fluctuations on spasticity, a mechanism associated with overactive spinal reflex. However, Sosnoff et al. [11] quantified muscle properties using electromyography (EMG) recordings, which is a limitation of this work since such recordings cannot directly evaluate muscular characteristics. Similarly, Shin and Sosnoff [12] tested whether Trembling fluctuations could reflect impairment levels in individuals with spinal cord injury (SCI) during sitting balance, with the underlying hypothesis that people with different levels of SCI are more spastic compared to the control group due to increased stiffness [13, 14]. However, there were no differences in Rambling and Trembling fluctuations between people with SCI compared to control subjects. However, muscle stiffness was not directly measured and the study was performed in sitting posture, which is fundamentally more stable compared to standing [15]. Therefore, it still remains unclear whether Rambling and Trembling fluctuations are related to muscle properties or whether lack of differences was due to different postural strategies in standing and sitting postures. Further work is therefore warranted to directly test whether Rambling and Trembling fluctuations are correlated to stiffness during standing balance control.

Tensiomyography (TMG), a non-invasive method for measuring muscle contractile properties including stiffness, can measure the overall muscular characteristics of a group of muscles in a particular limb, which can provide some insight on the influence of musculoskeletal properties on postural control. Specifically, TMG measures muscular mechanical responses based on radial belly displacement of the muscle caused by a single electrical stimulus [16, 17]. TMG was shown as an effective tool for evaluating mechanical properties of superficial skeletal muscles, including assessments of muscle stiffness and contraction time, which are related to postural control [18]. For example, TMG has been utilized to assess muscular conditions, effects of athletic training, and recovery after injury [19]. Specifically, TMG assessments reflect recruitment of different muscle fiber types [17]. It can also evaluate different muscle conditions, such as fatigue, enhancement or deconditioning after training or injury, temporal or morphological muscle synchronization, and detection of clinical lesions [20].

Therefore, this study aimed to verify whether the mechanical characteristics of skeletal muscles in the lower limbs, including stiffness properties and contraction time, are reflected in the Rambling and Trembling fluctuations during quiet standing. We first evaluated whether the mechanical properties of the skeletal muscles (stiffness and contraction time) are correlated to Rambling and Trembling fluctuations. Second, we tested whether stiffness and contract time of the individual muscles can be a predictor of postural control during upright posture. We hypothesized that muscle stiffness and contract time of each muscle, which are passive muscle properties, would: (i) be better correlated with Trembling fluctuations compared to Rambling fluctuations; and (ii) predict Trembling fluctuations components better compare to Rambling components. Affirmative answers to these questions would provide direct experimental evidence that the Trembling component, separate from the COP fluctuations, is associated to passive muscles properties, including stiffness, which is in support of the Rambling and Trembling hypothesis [4, 5].

## Materials and methods

### Participants

The experiment was conducted on 32 healthy young male volunteers. The mean (SD) age, weight and height were: 20.26 (1.67) years, 173.19 (5.44) cm, and 70.22 (8.96) kg, respectively. None of the participants had any history of serious injuries and neurological impairment as well as physical or mental illness in the past 6 months. All participants gave written informed consent in accordance with the principles of the Declaration of Helsinki. The study protocol was approved by the Yeungnam University institutional review board.

### Muscle stiffness and contraction time measurements

**Fig 1:**
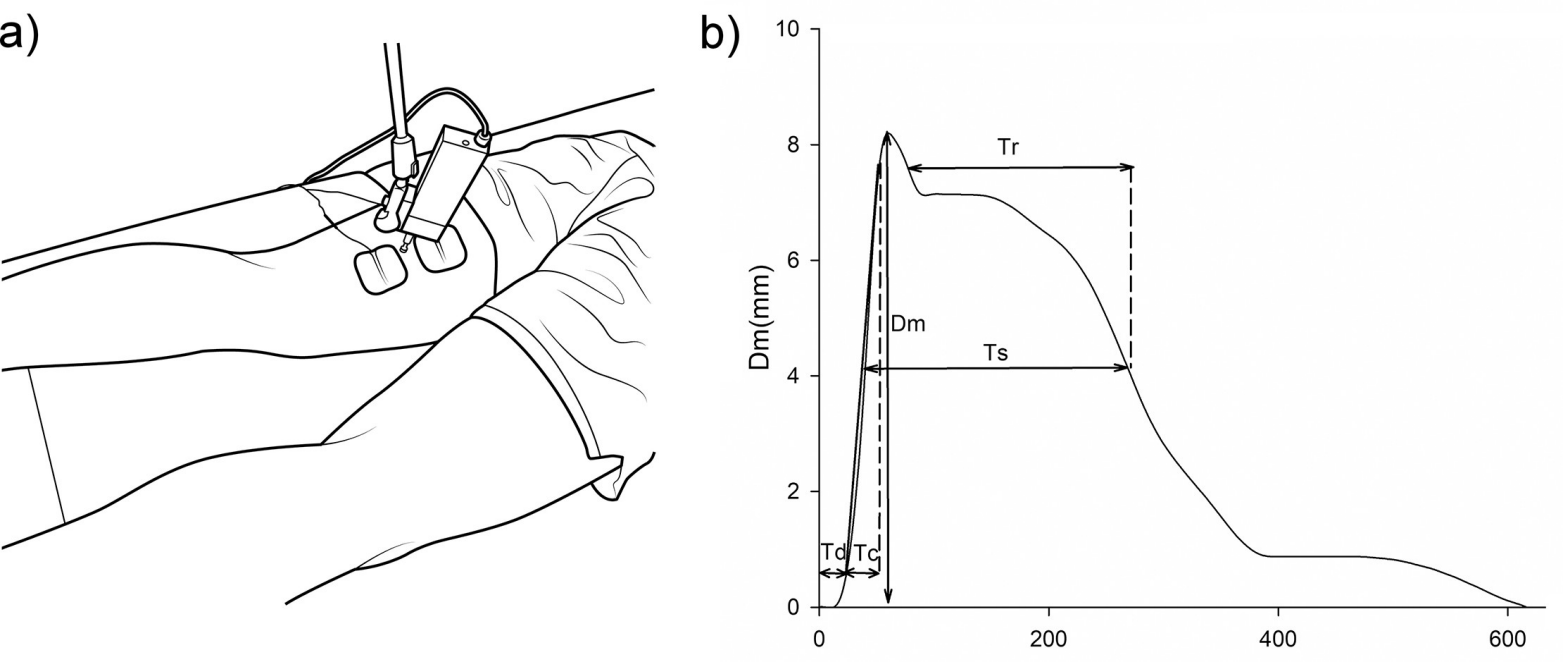
Tensiomyography (TMG) measurements: (a) Illustration of the experimental setup during of the measurement in the prone posture and (b) Sample typical evoked response during TMS measurements and an illustration of the extracted parameters: displacement of the muscle (Dm) and contraction time (Tc), as well as other parameters which were not evaluated in this study: relaxation time (Tr), sustain time (Ts), delay time (Td), and contraction velocity (Vc = (90%Dm—10%Dm) / Tc).

A tensiomyography system (TMG S1, TMG-BMC Ltd., Slovenia, EU) was used to measure muscle stiffness and contraction time of six muscles unilaterally on the right leg: (i) rectus femoris (RF), (ii) vastus medialis (VM), (iii) vastus lateralis (VL), (iv) biceps femoris (BF), (v) gluteus maximus (GT), and (vi) semitendinosus (ST). Tensiomyography is a non-invasive method for measurement of mechanical muscle contractile properties in response to a single electrical stimulus [16]. Two stimulating gel electrodes (ValuTrode, Axelgaard Manufacturing Co. Ltd., 5x5cm) were placed on each muscle separately, 3 cm apart (Fig 1A), to evoke contractions. Responses were evoked by applying a single monophasic electrical pulse, with a 1 ms pulse duration, at stimulation amplitudes between 40 and 100 mA. Specifically, the stimulation amplitude was varied from 40 to 100 mA, in 20 mA increments, while the evoked responses to each stimulus were monitored and recorded. The stimulation that evoked a maximal / plateau response (amplitude of evoked response remained unchanged when the stimulating current was increased) was then selected for to for the evoked responses. The stimulation intensity that evoked a plateau response always occurred at amplitudes above 80 mA (4 participants at 80 mA and 28 participants at 100 mA). A rest period of at least 10 sec between consecutive stimuli were used to prevent fatigue. During RF, VM, and VL measurements, subjects remained in the supine position, while a triangular wedge foam cushion was placed under the knee such that the knee joint angle remained at 120°. During BF, GT, and ST measurements, subjects remained in the prone posture, while a semicircular wedge foam cushion was placed on the ankle and the knee joint remained at 180° (Fig 1A). For each subject and each muscle, 5 evoked responses were obtained and averaged.

The evoked radial muscle belly displacement responses (Fig 1B) were recorded using the TMG data acquisition system at the sampling frequency of 1000 Hz. The plateau response was used to determine the maximum displacement of the muscle (Dm) (Fig 1B), which evaluates muscle stiffness [19]. Similarly, using the plateau response, the contraction time (Tc) was defined as the time from 10% to 90% of the muscle displacement curve (Fig 1B), which evaluates the rate of muscle contraction [16]. Other muscle contractile properties previously evaluated using TMG, including relaxation time (Tr), sustain time (Ts), delay time (Td), and contraction velocity (Vc = (90%Dm—10%Dm) / Tc) [16] (Fig 1B), were not include as they had no relation to the framework of this study.

### Postural control measurements

During postural control measurements, subjects were asked to maintain static upright posture in three different conditions: (a) eyes open, (b) eyes closed, and (c) foam standing (with eyes open), each lasting for 60 seconds. A rest period of at least 30 sec between consecutive conditions were used to prevent fatigue, and the order of conditions was randomized between participants. During each trial, participants stood in a natural upright posture with their feet positioned in a comfortable and natural position at shoulder width, and they were asked to gaze at a fixed target, at eye level, 2 m in front of them. For each 60 sec trial, data was analyzed in the 30 sec stable posture windows, which was chosen 5 sec after start of the trial [21].

**Fig 2:**
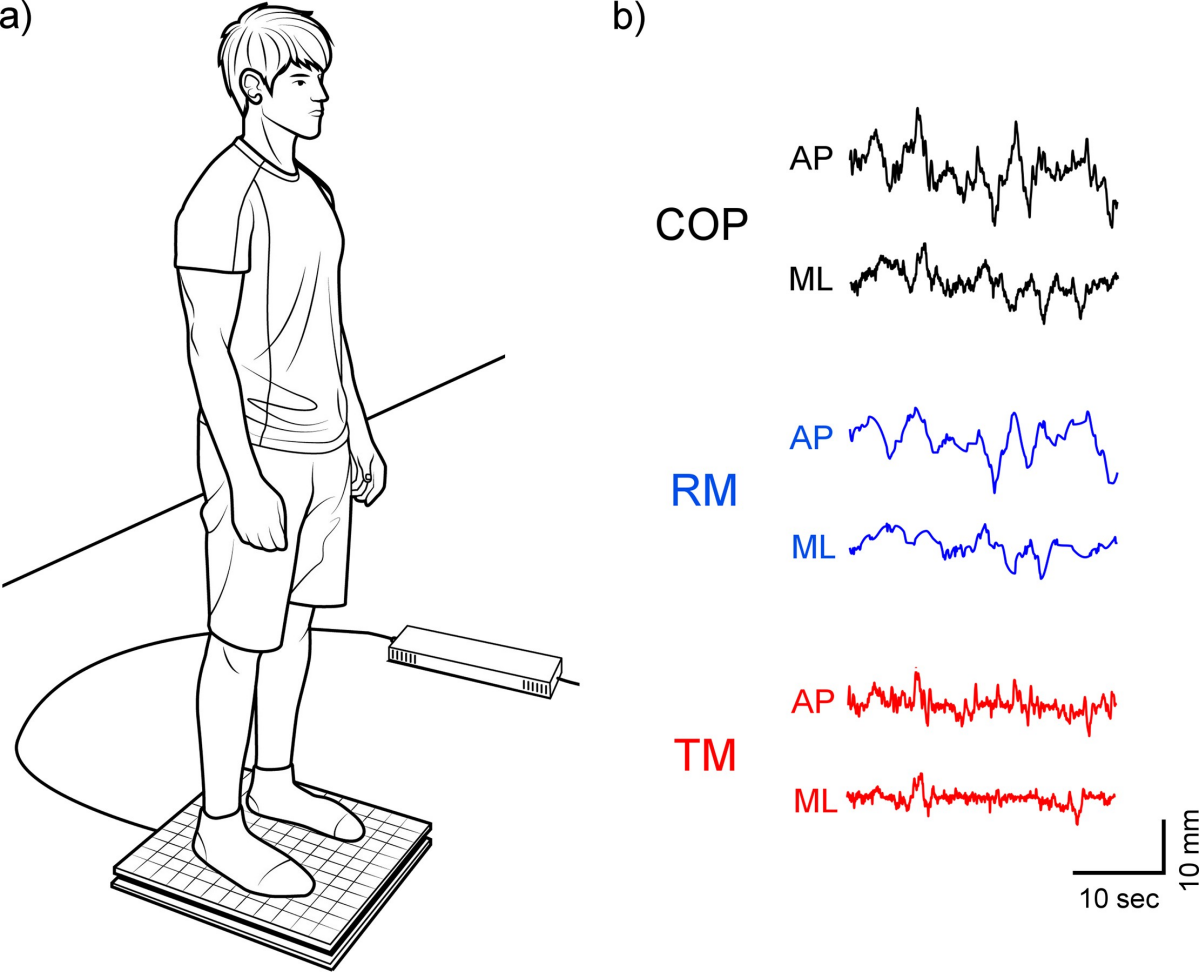
a) Illustration of the experimental setup during postural control measurements; and b) A representative sample of center of pressure (COP) trajectory as well as the rambling (RM) and trembling (TM) fluctuations decomposition from the 30s recording of the in the anterior-posterior (AP) and medial-lateral (ML) directions.

During the trials, subjects stood on a force plate (Accusway, AMTI, Watertown, MA, USA) (Fig 2A), which was used to measure forces (Fx, Fy, and Fz) and moments (Mx, My, and Mz). Data was recorded using a data acquisition system (PXIe-1078/6363, National Instruments, USA) at the sampling frequency of 100 Hz. The recorded force and moment time series were filtered using a fourth-order, low-pass Butterworth filter with a cutoff frequency of 10 Hz [11], and then the center of pressure (COP) was calculated to obtain the anterior-posterior (AP) and medial-lateral (ML) COP time series [2]. Next, each COP time series (COP_AP and COP_ML) was separated into the rambling (Rambling_AP and Rambling_ML) and trembling (Trembling_AP and Trembling_ML) components using a decomposition method described by Zatsiorsky and Duarte [5, 6]. In summary, the Rambling and Trembling decomposition method maintains the following relationship: COP = Rambling + Trembling. The Rambling component was determined by calculating the Instant Equilibrium Point (IEP) trajectory by: (1) identifying the zero-force points (i.e., ΣF_horizontal_ = 0, where ΣF_horizontal_ indicates the external forces acting on the body in the horizontal direction) and (2) interpolation of these points using a piecewise cubic Hermite polynomial method [12, 22]. The obtained trajectory is defined as the Rambling component, which represents the continuous fluctuation of the reference point on the supporting surface to maintain postural equilibrium [4, 5, 7]. The Trembling trajectory was calculated as the difference between the Rambling trajectory and the COP [4, 5]. Sample COP, Rambling, and Trembling components along the AP axis are illustrated in Fig 2B.

For each of the COP, Rambling, and Trembling time series in the AP and ML directions, time-domain parameters and frequency-domain parameters were calculated according to Prieto et al [23]. In summary, the time-domain parameters included: (a) mean distance (MD), which represents the average distance travelled by the COP (mm); (b) root mean square (RMS), which represents the standard deviation of the COP (mm); (c) range which the maximum distance between any two points on the COP path (mm); (d) mean velocity (MV), which represents the average velocity of COP (mm/s); (e) The 95% confidence circle area (CCA), which represents the area of a circle with a radius equal to the one-sided 95% confidence limit of the resultant distance time series(mm^2^); (f) 95% confidence ellipse area (CEA), which represents the area of the 95% bivariate confidence ellipse (mm^2^); (g) sway area (SA), which represents summing the area of the triangles formed by two consecutive points on the COP path and mean COP (mm^2^/s). The frequency-domain parameters, which were measured using Welch's power spectral density estimate method with a resolution of 0.033 Hz, included: (a) 50% power frequency (F50), which represents the median power frequency (Hz); (b) 95% power frequency (F95), which represents the frequency below which 95% of the total power is concentrated (Hz); (c) mean frequency (MF), which represents the average frequency (Hz); (d) total power (TP), which represents the integrated area of the power spectrum; (e) frequency at which the spectral mass is concentrated (CF), which represents the square root of the ratio of the second to the zeroth spectral moments (Hz); and (f) frequency dispersion (FD), which represents a unitless measure of the variability in the frequency content of the power spectral density. Each parameter was computed for the COP, ML, and Trembling time series for the AP and ML directions.

### Statistical analysis

One-way repeated measures analysis of variance (ANOVA) was used to compare changes in postural sway parameters during: (a) eyes open; (b) eyes closed; and (c) foam standing conditions. Significant results on the ANOVA test were followed up with post-hoc multiple comparisons with Holm adjustment [24]. Moreover, partial correlations and multiple linear regression stepwise analyses were performed to examine relationships between postural sway parameters (dependent variables: all time-domain and frequency-domain measures of COP, Rambling and Trembling fluctuations for AP and ML direction and muscle contractile properties (independent variables: Dm and Tc of six muscles). Specifically, the analysis controlled for the effects of age, height, weight, vision (i.e., corresponding postural sway parameter during eyes closed condition), and support surface (i.e., corresponding postural sway parameter during foam standing condition), while also applying the Holm adjustment [24]. Therefore, our analysis considers the effects of both vision and support surface during standing to provide an overall relationship between postural sway parameters and muscle contractile properties. All data were analyzed using SPSS 22 (IBM Inc., USA), and R 3.5.0. (R Core Team, 2018) [25]. Significance level was set at p < 0.006 after the Holm adjustment [24].

## Results

### Muscle contraction properties

The mean (SD) of contraction properties for all participants were: (i) RF Dm = 7.05 (2.25) and Tc = 25.31 (5.03); (ii) VM Dm = 6.82 (1.67) and Tc = 22.26 (4.05); (iii) VL Dm: 5.21 (1.55) and Tc = 22.55 (4.29); (iv) BF Dm = 3.91 (2.52) and Tc = 36.39 (19.25); (v) GT Dm = 7.31 (2.69) and Tc = 36.35 (8.97); and (vi) ST Dm = 6.15 (2.17) and Tc = 38.47 (10.34), where Dm was measured in mm and Tc in ms.

### Postural sway during eyes open, eyes closed, and foam standing

Comparison of postural sway parameters for the COP, Rambling, and Trembling fluctuations in the AP and ML directions between: (a) eyes open; (b) eyes closed; and (c) foam standing conditions are shown in Table 1. Most postural sway parameters in the eyes closed and foam standing conditions were significantly different from those in the eyes open condition (Table 1). Specifically, for the AP direction, the COP, Rambling, and Trembling amount of sway (Range_AP) was larger in eyes closed condition compared to eyes open condition, while the sway mean velocity (MV_AP) was faster in eyes closed condition compared to eyes open condition. For the ML direction, the COP, Rambling, and Trembling frequency domain parameters (F50_ML, F95_ML, MF_ML, TP_ML and CF_ML, except FD_ML) were larger in foam standing condition compared to both eyes open and eyes closed conditions. Since vision (i.e., eyes closed) and surface (i.e., foam standing) conditions affected postural control, these variables were included as control variables in the regression analysis.

**Table 1:** Comparison of time-domain and frequency domain parameters for the center of pressure (COP), rambling and trembling fluctuations in the anterior-posterior (AP) and medial-lateral (ML) directions during: eyes open; eyes closed; and foam standing conditions. Shown are the mean (SD) for each parameter.

		COP			Rambling			Trembling	
	Eyes open	Eyes closed	Foam standing	Eyes open	Eyes closed	Foam standing	Eyes open	Eyes closed	Foam standing
**MD_AP (mm)**	3.09(1.22)	4.39(3.23)	5.47(2.45)*	2.76(1.30)	3.58(2.52)	4.69(2.23)*	1.11(0.76)	1.65(0.78)*	1.77(0.58)*
**MD_ML (mm)**	1.01(0.39)	1.19(0.55)	2.88(10.6)*†	0.84(0.40)	1.02(0.55)	2.43(1.05)*†	0.50(0.27)	0.60(0.29)	1.14(0.53)*†
**RMS_AP (mm)**	3.82(1.45)	5.33(3.50)	6.71(2.80)*	3.37(1.54)	4.28(2.77)	5.65(2.55)*	1.59(0.93)	2.38(1.02)*	2.57(0.80)*
**RMS_ML (mm)**	1.27(0.46)	1.50(0.66)	3.54(1.18)*†	1.05(0.48)	1.24(0.61)	2.96(1.15)*†	0.74(0.35)	0.88(0.38)	1.66(0.68)*†
**Range_AP (mm)**	18.61(6.91)	25.49(10.62)*	31.34(9.57)*†	14.27(6.47)	18.56(9.71)	23.32(8.21)*	10.98(4.54)	16.29(5.68)*	18.45(5.56)*
**Range_ML(mm)**	7.13(2.23)	8.75(3.71)	17.67(5.10)*†	5.20(1.97)	5.95(2.32)	13.00(4.91)*†	5.98(2.55)	7.15(2.70)	12.26(4.03)*†
**MV_AP (mm/s)**	11.61(3.02)	15.85(4.59)*	17.37(3.58)*	3.75(1.20)	4.99(1.60)*	5.85(1.99)*	17.34(4.80)	23.22(6.80)*	25.96(5.59)*
**MV_ML (mm/s)**	9.52(3.63)	10.29(3.82)	13.08(3.61)*†	2.48(0.93)	2.84(1.15)	4.50(1.76)*†	14.25(6.00)	15.47(6.15)	19.10(5.48)*†
**CCA(mm**^**2**^**)**	171.57(117.26)	272.12(224.98)*	529.77(348.94)*	136.90(127.86)	211.61(341.81)	377.74(274.02)*	41.24(51.55)	79.39(66.06)*	103.53(58.63)*
**CEA (mm**^**2**^**)**	95.96(59.0)	165.62(161.49)*	461.32(269.51)*†	69.95(49.9)	110.43(109.72)	325.76(214.44)*†	24.68(24.6)	42.70(35.4)	84.79(52.61)*†
**SA (mm**^**2**^**/S)**	11.55(6.51)	17.49(9.77)	31.81(12.54)*†	2.73(1.34)	4.35(2.98)	9.41(5.49)*†	4.46(3.37)	6.92(4.17)*	10.50(4.50)*†
**F50_AP (Hz)**	0.23(0.06)	0.27(0.07)	0.25(0.06)	0.20(0.03)	0.21(0.03)	0.21(0.03)	0.56(0.28)	0.53(0.25)	0.58(0.19)
**F50_ML (Hz)**	0.48(0.23)	0.47(0.22)	0.30(0.10)*†	0.28(0.11)	0.26(0.10)	0.22(0.04)	0.73(0.26)	0.69(0.24)	0.55(0.22)
**F95_AP (Hz)**	0.92(0.33)	0.99(0.33)	0.91(0.32)	0.46(0.14)	0.49(0.11)	0.45(0.12)	1.68(0.61)	1.61(0.51)	1.73(0.54)
**F95_ML (Hz)**	1.70(0.88)	1.67(0.72)	0.90(0.27)*†	1.04(0.50)	0.99(0.54)	0.56(0.17)*†	2.46(0.87)	2.27(0.73)	1.67(0.66)*†
**MF_AP(Hz)**	0.70(0.35)	0.69(0.24)	0.59(0.24)	0.25(0.12)	0.27(0.11)	0.23(0.10)	3.07(1.37)	2.61(1.17)	2.53(0.89)
**MF_ML(Hz)**	1.72(1.04)	1.61(0.93)	0.80(0.35)*†	0.56(0.31)	0.52(0.28)	0.32(0.13)*†	5.03(1.90)	4.47(1.57)	3.11(1.35)*†
**TP_AP**	270.05(199.28)	645.71(1244.34)	863.86(729.71)	210.84(217.88)	380.38(647.47)	598.46(545.15)	62.43(87.88)	121.81(110.59)*	138.15(90.41)*
**TP_ML**	31.32(21.95)	47.34(49.82)	243.10(188.97)*†	21.62(20.71)	31.55(34.65)	164.73(153.54)*†	12.57(13.18)	17.58(17.96)	63.23(58.21)*†
**CF_AP (Hz)**	0.44(0.13)	0.48(0.13)	0.46(0.14)	0.27(0.07)	0.28(0.06)	0.25(0.06)	0.86(0.29)	0.83(0.26)	0.88(0.22)
**CF_ML (Hz)**	0.83(0.30)	0.81(0.28)	0.49(0.12)*†	0.50(0.21)	0.48(0.21)	0.31(0.08)*†	1.17(0.32)	1.10(0.26)	0.85(0.27)*†
**FD_AP**	0.75(0.04)	0.72(0.06)	0.73(0.05)	0.67(0.07)	0.64(0.07)	0.63(0.07)	0.66(0.10)	0.66(0.08)	0.64(0.09)
**FD_ML**	0.71(0.07)	0.72(0.06)	0.70(0.07)	0.73(0.05)	0.74(0.06)	0.66(0.07)*†	0.66(0.09)	0.65(0.09)	0.64(0.11)

*significant difference between eyes open and eyes closed conditions and between eyes open and Foam standing conditions

†significant difference between the Eyes closed and Foam standing conditions. Statistical comparisons were performed with Holm adjustment and significance level was set at p < 0.006 after the Holm adjustment [24].

### Partial correlations between muscle properties and postural sway

**Fig 3:**
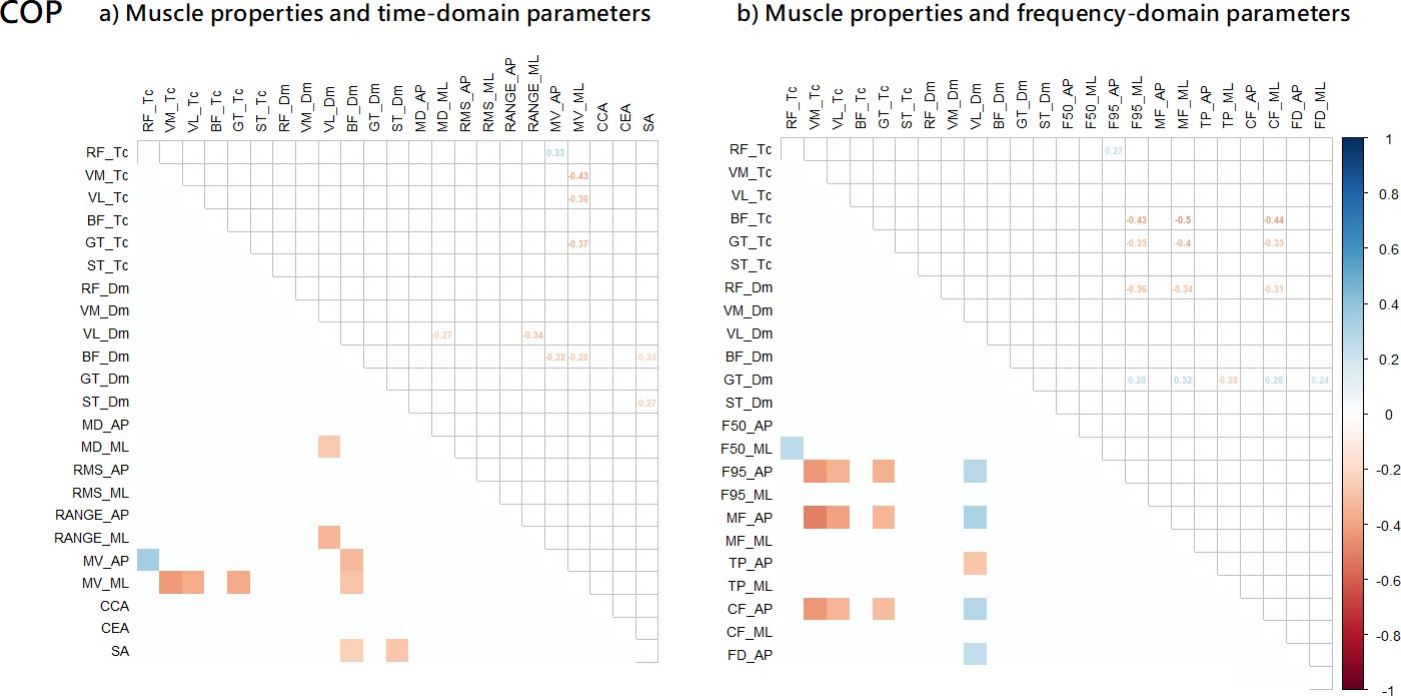
a) Partial correlation between the muscle properties and COP: a) time-domain parameters; and b) frequency-domain parameters.

The COP fluctuation partial correlation results are shown in Fig 3. Overall, Tc and Dm of the six muscles were associated with a total of 9 time-domain parameters (Fig 3A) and 14 frequency-domain parameters (Fig 3B). For time-domain parameters, contraction time (Tc) of the GT, VL, and VM muscles was negatively correlated (r range: -0.37 to -0.43) with the sway mean velocity (MV_ML), while stiffness (Dm) of the BF and VL was negatively correlated (r range: -0.24 to -0.34) with the amount of sway (Range_ML), the variability of sway (RMS_ML), the sway velocity (MV_AP and MV_ML), and the sway area (SA). Moreover, for frequency-domain parameters, contraction time (Tc) of the GT, VL, and VM muscles was negatively correlated (r range: -0.31 to -0.50) with the mean frequency, and centroidal frequency (F95_ML, MF_ML, and CF_ML), while stiffness (Dm) of the VL muscle was positively correlated (r range: 0.29 to 0.34) with the mean frequency, centroidal frequency, and frequency dispersion (F95_ML, CF_ML, and FD_ML).

**Fig 4:**
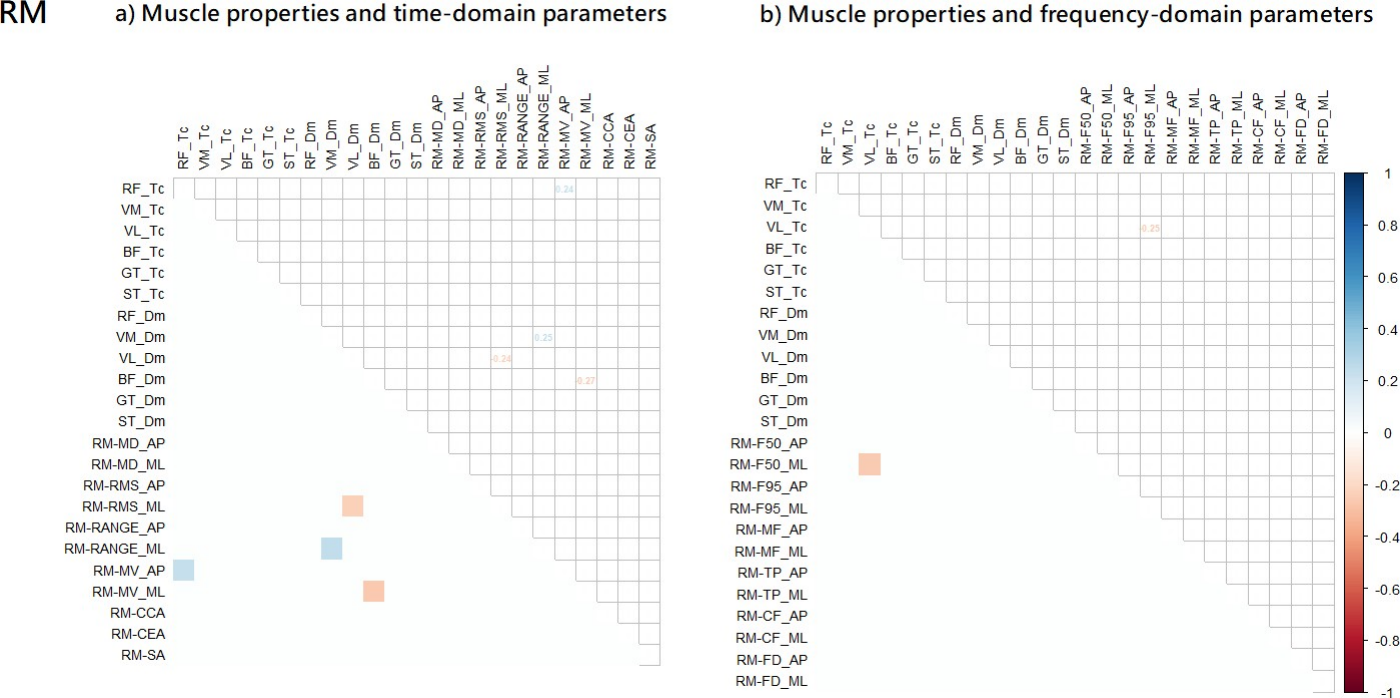
a) Partial correlation between the muscle properties and Rambling (RM): a) time-domain parameters; and b) frequency-domain parameters.

The Rambling fluctuation partial correlation results are shown in Fig 4. Overall, Tc and Dm of four muscles were associated with four time-domain parameters (Fig 4A) and one frequency-domain parameters (Fig 4B). For time-domain parameters, contraction time (Tc) of the RF muscle, and stiffness (Dm) of VM was positively correlated (r: 0.24, and 0.25) with the sway velocity (MV_AP) and the amount of sway (Range_ML), while stiffness (Dm) of BF, and VL muscles was negatively correlated (r: -0.24, and -0.27) with the sway velocity (MV_ML) and variability of sway (RMS_ML). Moreover, for frequency-domain parameters, contraction time (Tc) of the VL muscles was negatively correlated (r: 0.25) with mean frequency (F50_ML).

**Fig 5:**
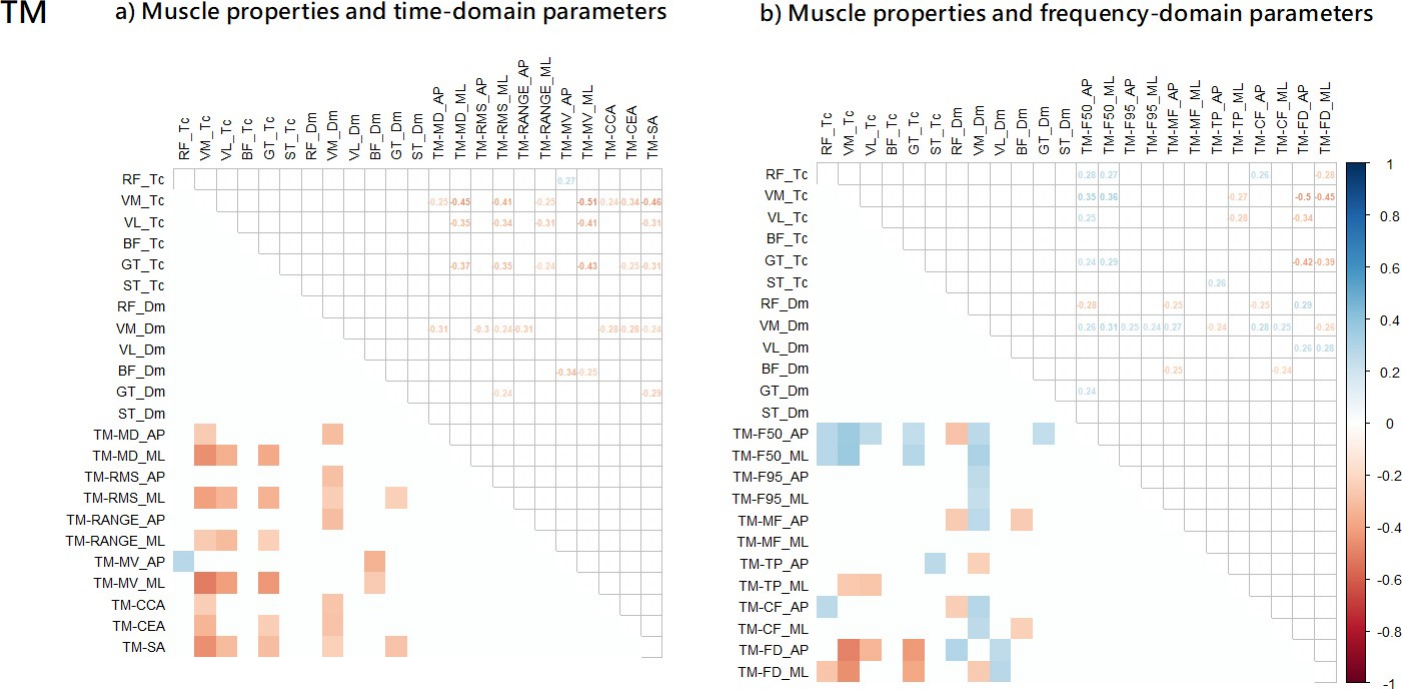
a) Partial correlation between the muscle properties and Trembling (TM): a) time-domain parameters; and b) frequency-domain parameters.

The Trembling fluctuation partial correlation results are shown in Fig 5. Overall, Tc and Dm of six muscles were associated with 31 time-domain parameters (Fig 5A) and 37 frequency-domain parameters (Fig 5B). For time-domain parameters, contraction time (Tc) of the BF, GT, VL, and VM muscles was negatively (r range: -0.25 to -0.51) correlated with all time-domain parameters (except for the RMS_AP and Range_AP), while stiffness (Dm) of the BF, GT, and VM was negatively correlated (r range: -0.24 to -0.34) with the amount of sway (MD_AP, and Range_AP), the variability of sway(RMS_AP, and RMS_ML), the sway velocity (MV_AP and MV_ML), and the sway area (CCA, CEA, and SA). Moreover, contraction time (Tc) of the GT, RF, ST, VL, and VM muscles was correlated (r range: -0.50 to 0.36) with all frequency-domain parameters (except for the F95_AP, F95_ML, MF_AP, MF_ML, and CF_ML), while stiffness (Dm) of BF, GT, RF, VL, and VM muscles was correlated (r range: -0.40 to 0.43) with all frequency-domain parameters (except for MF_ML).

### Prediction of muscle properties from postural sway parameters

Stepwise linear regression analyses revealed that both the Tc and Dm were significant predictors of variables of several time-domain and frequency-domain parameters of the COP, Rambling, and Trembling fluctuations, as indicated in Table 2 and Table 3. However, predictors of these parameters were different among COP, Rambling, and Trembling fluctuations.

**Table 2:** Summarized results of the multiple linear regression analysis of the time-domain parameters.

Dependent variables		COP			Rambling			Trembling	
	R^2^	Predictors	Sig	R^2^	Predictors	Sig	R^2^	Predictors	Sig
**MD_AP**	0.141	Surface	0.000	0.106	Surface	0.001	0.361	Surface	0.000
		Vision	0.036					Vision	0.001
								GT_DM	0.020
								ST_TC	0.000
								BF_DM	0.001
								ST_DM	0.005
								GT_TC	0.008
**MD_ML**	0.573	Surface	0.000	0.491	Surface	0.000	0.504	Surface	0.000
								GT_TC	0.000
								ST_TC	0.001
								ST_DM	0.017
**RMS_AP**	0.162	Surface	0.000	0.119	Surface	0.001	0.267	Surface	0.000
		Vision	0.030					Vision	0.001
								GT_DM	0.008
		1						ST_TC	0.019
**RMS_ML**	0.647	Surface	0.000	0.539	Surface	0.000	0.541	Surface	0.000
		VL_DM	0.003					GT_TC	0.000
		Age	0.015					ST_TC	0.001
								ST_DM	0.012
**Range_AP**	0.253	Surface	0.000	0.172	Surface	0.000	0.267	Surface	0.000
		Vision	0.003		Vision	0.040		Vision	0.000
**Range_ML**	0.644	Surface	0.000	0.53	Surface	0.000	0.513	Surface	0.000
		VL_DM	0.001					GT_TC	0.000
		Age	0.002					ST_TC	0.002
								ST_DM	0.021
**MV_AP**	0.498	Surface	0.000	0.312	Surface	0.000	0.467	Surface	0.000
		Vision	0.000		Vision	0.002		Vision	0.000
		RF_TC	0.000		RF_TC	0.009		BF_DM	0.000
		BF_DM	0.000		BF_DM	0.016		RF_TC	0.000
**MV_ML**	0.303	Surface	0.000	0.394	Surface	0.000	0.444	VL_TC	0.002
		GT_TC	0.000		BF_DM	0.001		Surface	0.000
		BF_DM	0.014		GT_DM	0.014		GT_TC	0.000
								ST_TC	0.006
								ST_DM	0.001
								Weight	0.002
								RF_TC	0.029
**CCA**	0.094	Surface	0.002	0.113	Surface	0.001	0.415	Surface	0.000
								Eyes-closed	0.012
**CEA**	0.096	Vision	0.029	0.384	Surface	0.000	0.431	Surface	0.000
								GT_TC	0.000
								ST_TC	0.000
								ST_DM	0.01
								Vision	0.048
**SA**	0.114	Vision	0.016	0.386	Surface	0.000	0.502	Surface	0.000
					RF_DM	0.049		GT_TC	0.000
								ST_TC	0.000
								ST_DM	0.000
								Vision	0.005
								VL_TC	0.049

**Table 3:** Summarized results of the multiple linear regression analysis of the frequency-domain parameters.

Dependent variables		COP			Rambling			Trembling	
	R^2^	Predictors	Sig	R^2^	Predictors	Sig	R^2^	Predictors	Sig
**F50_AP**	0.044	Vision	0.040			n.s	0.277	RF_TC	0.001
								RF_DM	0.001
								VL_TC	0.005
								Height	0.021
								DF_DM	0.043
**F50_ML**	0.209	Surface	0.000	0.054	Surface	0.022	0.471	Surface	0.000
		VL_TC	0.013					GT_TC	0.000
								ST_TC	0.000
								ST_DM	0.000
								BF_DM	0.000
								RF_TC	0.001
**F95_AP**	0.075	RF_TC	0.007	0.041	Vision	0.047	0.308	RF_TC	0.001
								BF_DM	0.000
								GT_DM	0.008
								ST_TC	0.000
								BF_TC	0.004
								ST_DM	0.013
**F95_ML**	0.441	Surface	0.000	0.277	Surface	0.000	0.157	Surface	0.000
		VL_TC	0.000		VL_TC	0.010			
		Weight	0.006		Weight	0.032			
		RF_DM	0.023						
**MF_AP**			n.s			n.s	0.296	RF_TC	0.006
								BF_DM	0.000
								GT_DM	0.000
								ST_TC	0.002
								BF_TC	0.004
**MF_ML**	0.454	Surface	0.000	0.220	Surface	0.000	0.187	Surface	0.000
		VL_TC	0.000		Weight	0.044			
		RF_DM	0.007		VL_TC	0.047			
		Weight	0.010						
**TP_AP**	0.049	Surface	0.030	0.075	Surface	0.007	0.194	ST_TC	0.007
								Surface	0.002
								Vision	0.012
								GT_DM	0.042
**TP_ML**	0.454	Surface	0.000	0.342	Surface	0.000	0.359	Surface	0.000
		VL_DM	0.028					GT_TC	0.003
								ST_TC	0.039
**CF_AP**	0.059	RF_TC	0.017			n.s	0.324	RF_TC	0.000
								RF_DM	0.092
								BF_DM	0.000
								ST_TC	0.000
								ST_DM	0.000
								GT_TC	0.024
**CF_ML**	0.466	Surface	0.000	0.284	Surface	0.000	0.261	Surface	0.000
		VL_TC	0.000		VL_TC	0.013		BF_DM	0.008
		Weight	0.012		Weight	0.036		GT_DM	0.025
		RF_DM	0.015						
**FD_AP**			n.s			n.s	0.415	GT_TC	0.000
								RF_DM	0.003
								VL_TC	0.017
								ST_TC	0.000
								RF_TC	0.020
								BF_TC	0.009
								ST_DM	0.014
**FD_ML**			n.s	0.268	Surface	0.000	0.448	GT_TC	0.000
					VL_DM	0.015		BF_TC	0.001
								RF_TC	0.003
								ST_TC	0.000
								ST_DM	0.000
								Age	0.001
								VL_DM	0.001

COP fluctuation time-domain parameters indicate that Tc and Dm of all muscles, except for the VM and ST, were predictors in 4 linear regression models: RMS_ML, Range_ML, MV_AP, and MV_ML (Table 2), while COP fluctuation frequency-domain parameters indicate that Tc and Dm of RF, and VL muscles were predictors in 7 linear regression models: F50_ML, F95_AP, F95_ML, MF_ML, TP_ML, CF_AP, and CF_ML (Table 3).

Rambling fluctuations were not well predicted by the Tc and Dm of all six muscles as indicated by the time-domain and frequency-domain parameters. Rambling fluctuation time-domain parameters indicate that Tc and Dm of all muscles predicted three dependent variables (MV_AP, MV_ML, and SA_DM) (Table 2). Rambling fluctuation frequency-domain parameters indicate that Tc and Dm of all muscles predicted four dependent variables (F95_ML, MF_ML, CF_ML, and FD_ML) (Table 3).

Finally, Trembling fluctuations were best predicted by the Tc and Dm of all six muscles as indicated by the time-domain and frequency-domain parameters. Trembling fluctuation time-domain parameters indicate that Tc and Dm of all muscles predicted all dependent variables (except Ranage_AP, and CCA) (Table 2). Trembling fluctuation frequency-domain parameters indicate that Tc and Dm of all muscles predicted all dependent variables (except F95_ML, and MF_ML) (Fig 3).

## Discussion

Standing postural control in maintained through integration of multiple sensory systems (i.e., visual, vestibular, and somatosensory) through muscle activations and passive dynamics (i.e., ligament, joint, and muscle stiffness) within the CNS [1]. To understand these processes, the COP fluctuations can be separated into Rambling and Trembling components [5, 6]. These components are thought to be controlled through two independent processes: supraspinal contribution (Rambling component) and mechanical properties, including reflex control as well as joint, ligament, and muscle stiffness (Trembling component) [5, 6]. Previously, stiffness properties were evaluated during upright standing, which may involve both joint and ligament as well as muscle properties [18], while our current study evaluated stiffness in the prone posture which reflects mechanical muscle properties more accurately. Specifically, this study sought to test the Rambling and Trembling hypothesis by examining the relationship between mechanical muscle properties of lower limb skeletal muscles and postural fluctuations. We hypothesized that stiffness (i.e., Dm) and contraction time (i.e., Tc) of lower limb muscles would be better correlated as well as be a better predictors of Trembling fluctuations compared to the Rambling fluctuations [5, 6]. In this study, we showed that: (1) muscle stiffness (i.e., Dm) and contraction time (i.e., Tc) were better correlated with the time-domain and frequency-domain parameters of Trembling component compared with those of Rambling component; (2) Dm and Tc also predicted the time-domain and frequency-domain parameters of the Trembling component better compared to the Rambling component. To our knowledge, the current study is the first study to directly demonstrate the role of muscle properties in postural control using the Rambling and Trembling analysis method. Specifically, our work demonstrated an alternative approach for studying muscle stiffness (and other muscle property) contributions, which can offer complementary interpretations to understanding stiffness during quiet standing to the work of Winter et al. [26], Collins et al. [27], and Loram and Lakie [28].

First, our analysis demonstrated that muscle properties, including muscle stiffness and contraction time had a stronger relationship with the Trembling component, which are expected to be more related to muscle stiffness and other spinal reflex mechanisms compared to Rambling component. As hypothesized, this finding shows that the mechanical characteristics of skeletal muscles in the lower limbs, including stiffness properties and contraction time, are reflected adequately in the Trembling fluctuations during quiet standing. Specifically, the contractile properties of each muscle were more correlated with Trembling than with Rambling, while COP fluctuations were somewhat correlated. Muscle stiffness (Dm) and muscle contraction time (Tc) showed some correlations with COP fluctuations (time-domain: 6.8% and frequency-domain: 10.4%) parameters (Fig 3). Interestingly, after the Rambling and Trembling decomposition, muscle stiffness (Dm) and muscle contraction time (Tc) were more correlated with Trembling fluctuations (time-domain parameter: 23.5% and frequency-domain: 24.3%) than with Rambling fluctuations (time-domain parameter: 3.0% and frequency-domain: 0.7%) (Fig 4 and Fig 5). Moreover, results from this study, in which the correlation between muscle contractile properties and Trembling showed an overall negative tendency for both muscle stiffness (Dm) and contraction time (Tc) (Fig 5), suggest that individual with higher muscle stiffness (i.e. lower Dm) and shorter contraction time swayed more and faster according to the Trembling fluctuations component during standing. This implies that adequate muscle properties are a prerequisite for the stable standing posture. Future work should identify optimal and minimal muscle contractile properties in people with neurological disorders associated with degenerated postural control.

Second, our results showed that muscle contractile properties predict Trembling fluctuations better compared to Rambling in the regression models. In the regression models of the time- and frequency-domain parameters of COP fluctuations, muscle stiffness (Dm) and muscle contraction time (Tc) (except for the VM and GT) were included in a total of 11 linear regression models. After the decomposition, in the regression models of the time- and frequency-domain parameters of Trembling fluctuations, muscle stiffness (Dm) and muscle contraction time (Tc) of all six muscles became significantly better predictors compared to Rambling fluctuations. Specifically, muscle stiffness (Dm) and muscle contraction time (Tc) were involved in several more multiple linear models in case of Trembling, compare to Rambling (19 vs. 7, respectively) (Table 2 and Table 3). These results, which showed that Trembling had more predictors compared to Rambling and COP fluctuations, imply that the relative contribution of muscle contractile properties in postural control could be masked by simply analyzing COP fluctuations [2]. Nevertheless, the association of the major muscles with Tc and Dm was not completely excluded from Rambling parameters (Table 2 and Table 3), possibly because they include the results of the interaction between the CNS and muscle stiffness that are not distinguished simply by the Rambling component. This suggests a possible limitation of the Rambling and Trembling method.

Third, the results of this study also showed that muscle stiffness (Dm) and contraction time (Tc), measured in the prone position to evaluate mechanical muscle properties independent of the CNS contribution (e.g., spinal reflex contribution) and ligament and joint stiffness contributions, which are more present during upright standing, were still related to postural control. Zatiorskiy and Duarte [5, 6] proposed that the Trembling fluctuation would be affected by both the spinal reflexes and change in the intrinsic mechanical properties of the muscles and joints or ligament [8]. However, previous studies were limited in identifying the contributions of Trembling component to postural control [12, 22]. For instance, Shin and Sosnoff [12] could not demonstrate Trembling contributions during sitting balance control in people with SCI and compared to the able-body group since muscle stiffness was not directly measured. Moreover, a recent study reported that contractile properties of the gastrocnemius muscle, including muscle tone, elasticity, and stiffness changed depending on the posture assumed (i.e., increased contributions during unstable upright posture compare to stable as well as prone postures). Upright posture is maintained by superimposing different body parts (i.e., head, body, or lower limbs) along the longitudinal axis [29, 30], which requires postural muscle tone [31]. Therefore, muscle stiffness during prone posture, which was evaluated in present study, should be somewhat differently interpreted than muscle stiffness previously measured during standing which includes postural tone [18, 29, 30]. Our findings provide evidence that pure muscle stiffness (Dm) and contraction time (Tc), free of contributions of reflex control and joint / ligament influence, still partially represent the Trembling component which were derived from COP fluctuations. Therefore, these findings suggest that the posture tends to be partially controlled pure muscle properties of the lower limbs, rather than being fully dependent on the CNS and joint / ligament stiffness in standing posture.

Our results also revealed that lower limb muscle contractile properties of various hip and thigh muscle groups (i.e., rectus femoris, vastus medialis, vastus lateralis, biceps femoris, gluteus maximus, and semitendinosus) can affect postural control during standing. The results showed that many muscle contractile parameters still had greater correlations with the Trembling component (Fig 5). These results imply that Trembling as a summation results from the partial contributions of each local muscles. It is conceptually similar to results of the previous study about the effect of joint immobilization [8], which showed that Trembling fluctuation were increased only after knees, hips, and trunk were immobilization, implying that standing posture did not depend on one or two joints but summation of multi-joint cooperation of ankles, knees, and hips joints. On the contrary, inverted pendulum model of upright standing [2, 3] is mostly focused on the calf (gastrocnemius and soleus) muscle contributions to standing balance control in the AP direction [29, 32]. However, there are limitations to the inverted pendulum model in postural movements [8, 33–36]. For example, Pinter et. al [33] confirmed that variance of lower leg, upper leg, and head–arms–trunk segment contributes the postural control during unperturbed stance, rather than just the knees. Therefore, our finding also implies that various hip and thigh muscles play an important role in standing balance, which could provide evidence that the single link inverted pendulum model, which only considers calf muscle control of standing posture, should be expanded to include effect of various other muscles, perhaps via a multi-link inverted pendulum model [8, 29].

Our results also showed that the contribution of muscle contraction properties, as a predictor, were not different between AP and ML directions of postural fluctuation directions. Muscle stiffness (Dm) and muscle contraction time (Tc) were commonly predictors in all linear regression model of the time-domain parameters and some frequency-domain models (Table 2 and Table 3). Even though our results showed COP fluctuations in ML direction compared to the AP direction were small, after decomposition, muscle stiffness (Dm) and muscle contraction time (Tc), as predictors, in Trembling fluctuation in ML direction are still apparent. This implies that bi-directional contribution of Trembling is not casual. There are two possible explanations for the overall bi-directional influence on the Trembling fluctuations. First, muscle stiffness (Dm) and contraction time (Tc), estimated from of each muscles, is too limited to modulate posture with a distinct pattern [32, 37]. In addition, since the human body has multiple connections, it could be difficult to uncover a regular pattern in many muscles while standing naturally [37]. Further, standing posture is a complex process involving passive forces arising from the bones originating from the elongated ligaments, such as fully extended knee joints or ligaments in front of the hip, as well as viscoelastic properties of the muscles themselves, which can be influenced by the different physical characteristics of the subjects [4]. There could be a limit in concluding on the uniformly distributed constant role of each muscle in Trembling fluctuations because Trembling fluctuations are summation of muscles, joints, and ligaments contributions [5, 6].

## Conclusion

In this study, we investigated whether muscular stiffness (Dm) and muscle contraction (Tc) were related to the characteristics of the Rambling and Trembling fluctuation components. Our results showed that muscle contractile properties during standing posture affect balance ability, and that the Rambling and Trembling components, derived from the measured COP fluctuations, can be a useful to reflect supraspinal and peripheral mechanisms of postural control system.

## Supporting information

S1 File COP, RM, TM and TMG data for all participants. (CSV)
